# Excellent thermoelectric properties induced by different contact geometries in phenalenyl-based single-molecule devices

**DOI:** 10.1038/s41598-017-11224-x

**Published:** 2017-09-07

**Authors:** Xuan-Hao Cao, Wu-Xing Zhou, Chang-Yong Chen, Li-Ming Tang, Mengqiu Long, Ke-Qiu Chen

**Affiliations:** 1 Department of Applied Physics, School of Physics and Electronics, Hunan University, Changsha, 410082 China; 2 Physics Institute, Shaoguan University, Shaoguan, 512005 China; 3 Hunan Key laboratory of Super Micro-structure and Ultrafast Process, Central South University, Changsha, 410083 China

## Abstract

We investigated the thermoelectric properties of phenalenyl-based molecular devices by using the non-equilibrium Green’s function method combined with density function theory. The results show that the thermoelectric performance of molecular device can be significantly improved by different contact geometries. The ZT value of the device can reach 1.2 at room temperature, which is two orders of magnitude higher than that of graphene. Moreover, the change of the coupling between molecule and electrodes can also enhance the ZT value. The ZT value can be further optimized to 1.4 at 300 K and 5.9 at 100 K owing to the decrease of electronic thermal conductance and almost unchanged power factor.

## Introduction

Thermoelectric materials have the advantage of realizing the mutual transformation of heat and electricity without moving parts or working fluids^1^. Although they are reliable, clean, environment-friendly and compact when compared with traditional thermal engine, its commercial applications is still limited due to the low thermoelectric conversion efficiency^1, 2^. The performance of the thermoelectric materials can be characterized by a unitless parameter, which is called figure of merit $ZT=\frac{\sigma {S}^{2}}{{\kappa }_{e}+{\kappa }_{ph}}T$. To be competitive with the conventional heat machines, the ZT value of a thermoelectric material should be larger than 3^3^. However, to optimize the ZT value is a difficult task, as the electrical conductivity *σ*, the Seebeck coefficient *S*, the phonon thermal conductivity *κ*_*ph*_, the electron thermal conductivity *κ*_e_ and the temperature *T* are fully coupled with each other^4^. For instance, *σ* and *κ*_*e*_ obey the Wiedemann-Franz law^5^. Two strategies have been chosen to improve the ZT value^6^. One approach is to search for electron-crystal−phonon-glass materials^7, 8^, whose electrical conductivity is similar to crystal while having a glass-like phonon thermal conductivity, for instance Core-shell Nanowires^9–11^ and superlattices^12, 13^. Another method is to enhance the Seebeck coefficient by the increase of the electrical density of states at the Fermi level in low-dimensional structures^14^, such as quantum rings^15^, quantum wires^16–19^, antidot arrays^20^, nanoribbons^21, 22^, nanotubes^23, 24^ and single-molecule devices^25, 26^. Graphene is a wonder material with remarkable electrical and thermal properties. What’s more, it has the advantages of elasticity, cheap, low weight, material abundance, and large-area deposition compared with inorganic thermoelectric materials. However, it is not initially expected to be a good candidate for thermoelectric application because its heat conductivity is as high as 5 *kW*/*mK*, which leads to its ZT value to be only 0.01^27, 28^. But recent computational works show that a high ZT values could be reached in modified graphene nanoribbons (GNRs) which have exploited with quantum coherence effects^29–35^. Accordingly, modified graphene nanoribbon has recently received attention as a potential thermoelectric material. By using modified GNRs as backbone, we can then design the device with higher thermoelectric performances.

Recently, Marius *et al*.^26^ systematically studied the thermoelectric properties of paracyclophane-based single-molecule junctions with gold electrodes by using a fully first-principles-based method, and find that it is possible to chemically adjust the transport properties and enhance the ZT value. Although in laboratory-based conductance measurements gold is widely employed as the electrode material, gold nanoelectrodes are unstable at room temperature^36^. Therefore, electrodes with nanometer gap have been proposed to use the sp2-bonded two-dimensional carbon-based material, GNRs^37^. In addition to its excellent stability and conductivity, another notable advantage of GNRs electrodes is that their Fermi energy is closed to the molecular orbitals of organic molecules when single-molecule junctions is formed. Furthermore, Fan *et al*.^38^ theoretically explored the electronic properties of phenalenyl molecular device with different contact geometries, but the systematic research of thermoelectric properties of these phenalenyl molecular devices has not been reported yet. In the present work, we investigate the thermoelectric properties of phenalenyl-based molecular devices with two kinds of zigzag GNRs electrodes using the non-equilibrium Green’s function method combined with the density functional theory. The computational results show that M1_1_ have the highest ZT value among M1_1_-M3_1_, which is mainly determined by the electrical transport properties of phenalenyl-based molecular devices, and their thermoelectric performance can be optimized by the coupling between the phenaleny molecule and zigzag GNRs leads.

## Results and Discussion

**Figure 1:**
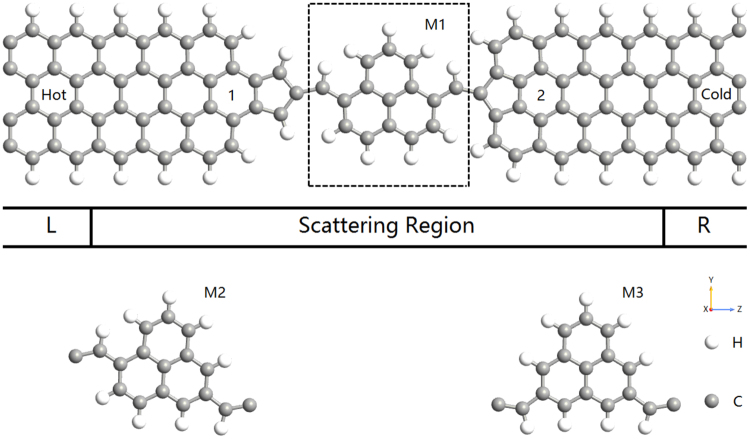
Configurations of M1_1(2)_-M3_1(2)_. The dashed box represents the replacement area of central molecule and the number 1(2) mean two different leads.

The molecular devices we simulated are illustrated in Fig. 1. We divided the whole system into three parts: left lead (hot bath), central region, and right lead (cold bath). For central phenalenyl molecule, which is a well-known stable organic radical with high symmetry, we bond it to two zigzag GNRs using penta-graphene. According to the different contact geometries between the molecule and electrodes, the devices can be divided into M1_1(2)_, M2_1(2)_ and M3_1(2)_. The subscript represents two types of penta-graphene structures, for example, M1_2_ indicates that the central molecule of device is M1 and the left and right leads are both type-2 electrodes, as shown in Fig. 1. The initial distance between two GNRs has been optimized. Each electrode has been described as a supercell which contains two repeated unit cells along the transport direction. Both the edge of the leads and the scattering region are hydrogenated. The thermoelectric properties of zigzag GNRs with 4 carbon dimer lines crossing the width (4ZGNR) have also been studied for comparison.

**Figure 2:**
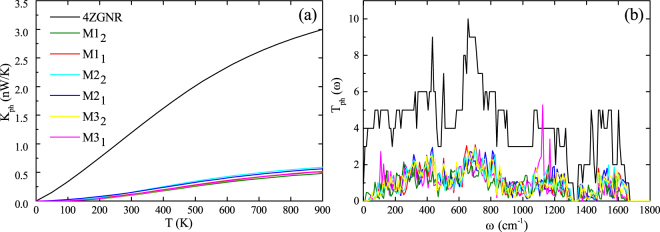
(**a**) The temperature-dependent phonon thermal conductance of all devices. (**b**) The phonon transmission spectrum of all models.

The calculated phonon thermal conductance and phonon transmission functions for 4ZGNR and M1_1(2)_-M3_1(2)_ are illustrated in Fig. 2(a) and (b). In the Fig. 2(a), we can clearly see that the phonon thermal conductance of molecular devices will be largely reduced by introducing the phenalenyl molecule when compared with 4ZGNR. What’s more, from Fig. 2(b), we can observe that the phonon transmission functions are restrained for our molecular configurations, which means the scattering from central region will affect both the lower acoustic and the higher optical phonons significantly. This results in the dramatically decrease of phonon thermal conductance. Nevertheless, we can also find that the discrepancy of the phonon transmission function of these molecular models is not obvious, indicating that the different contact geometries will not obviously influence their vibrational modes. As the temperature rises, gradually excited high frequency phonon modes will not significantly expand these differences. Therefore, although the difference increases with the increase of the temperature, the difference between the phonon thermal conductance of the six molecular devices is rather small in all the temperatures we considered. Thus, the difference of the thermoelectric performance by phonon thermal conductance is also small.

**Figure 3:**
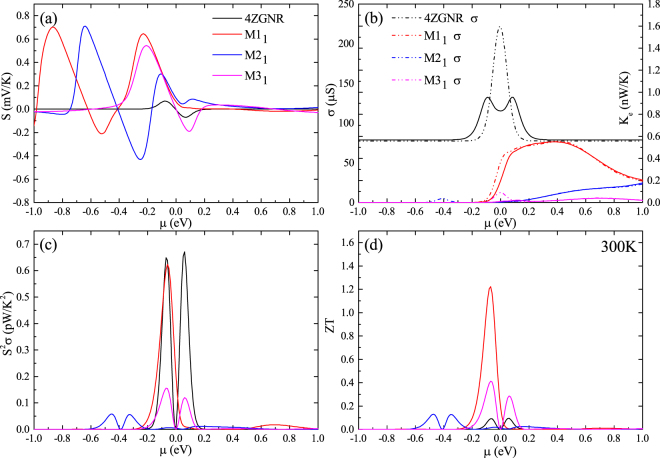
(**a**) Seebeck coefficient, (**b**) electronic conductance and electronic thermal conductance, (**c**) power factor. (**d**) Figure of merit ZT of M1_1_-M3_1_ and 4ZGNR as a function of chemical potential at room temperature.

To reveal the effects of the different contact geometries on the thermoelectric properties of phenalenyl-based molecular junctions, we computed the electronic properties of M1_1_-M3_1_ and 4ZGNR. Figure 3(a–c) show the Seebeck coefficient *S*, power factor *S* ^2^ *σ*, electrical conductance *σ* and electrical thermal conduction *κ*_e_ of the four configurations at room temperature, respectively. In general, we can identify that the electronic properties of the devices can be markedly modified by changing the type of the connection geometry between electrodes and molecule. Furthermore, compared to M2_1_ and M3_1_, M1_1_ have admirable power factor which can rival to 4ZGNR (see Fig. 3(c)). Fortunately, as shown in Fig. 3(b), unlike the extremely high electron conductance of 4ZGNR, the electronic thermal conductance of M1_1_ has been significantly reduced. In addition to the phonon properties studied above, the reduced thermal conductance can notably enhance the thermoelectric efficiency of M1_1_ when compared with 4ZGNR. The cases of M2_1_ and M3_1_ are different from M1_1_. We can find from Fig. 3(a–c) that the electrical conductance is fallen much, while the Seebeck coefficient have almost the same value of M1_1_ at the point of chemical potential where power factor obtains the maximum value, leading to the aggravation of power factor of M2_1_ and M3_1_. Therefore, from the discussion above, we can know that the electronic conductance is the main factor which leads to the large differences in the power factor of the three molecular models.

**Figure 4:**
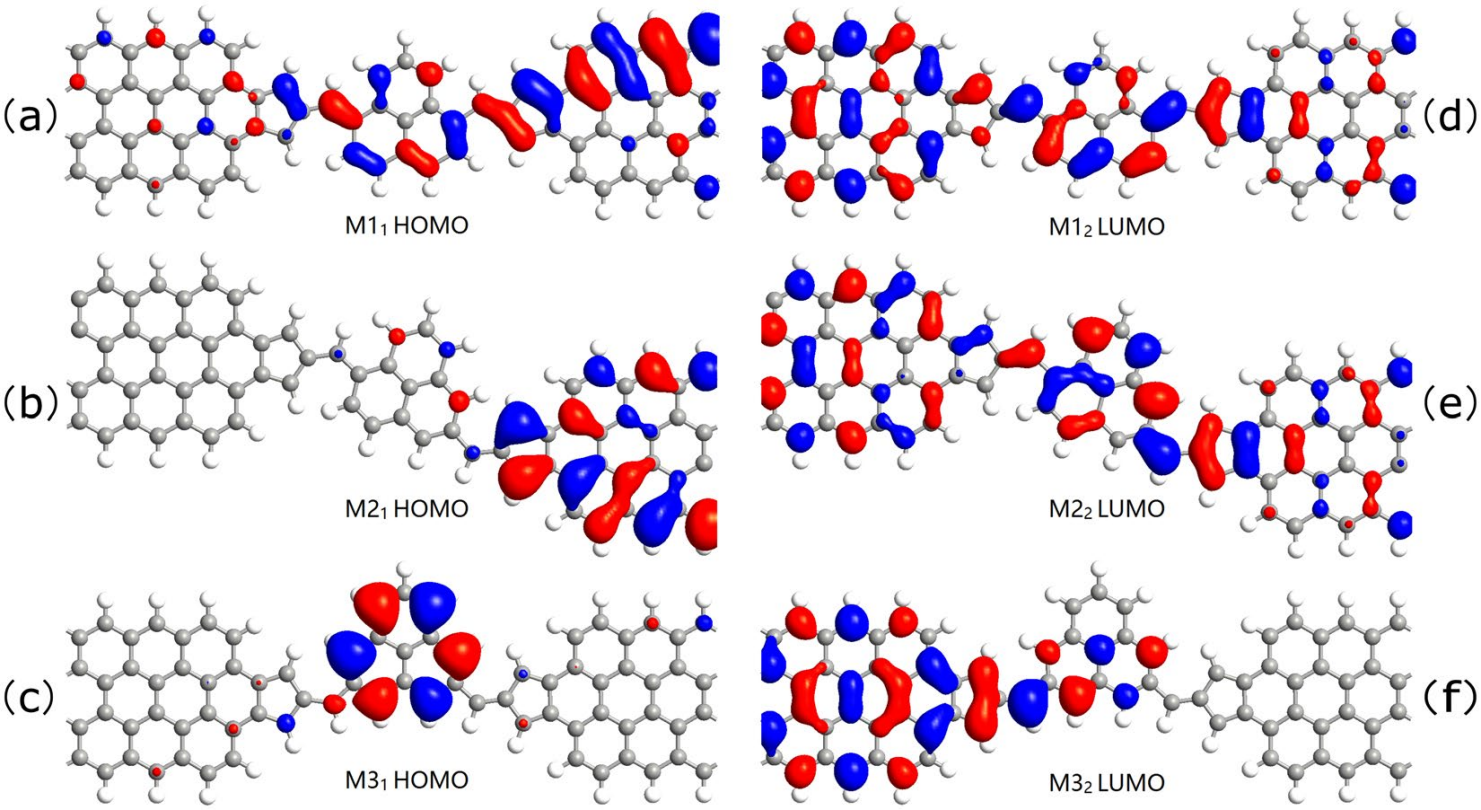
MPSH of the highest occupied molecular orbital and the lowest unoccupied molecular orbital for the central region of M1_1_-M3_1_ at the zero bias.

To trace the origin of these phenomena, we obtain the molecular projected self-consistent Hamiltonian (MPSH) energy spectrum of the two frontier molecular orbitals, which are called HOMO (highest occupied molecular orbital) and LUMO (lowest unoccupied molecular orbital) of the three molecular configurations (Fig. 4). In molecular electronics, we know that the electron transport mainly depends on the frontier molecular orbitals. Because the HOMO and LUMO are arranged on the sides of the nearest location of Fermi level, so they are of significant influence on the electronic transport of molecular devices. Furthermore, spatial distributions of orbitals, the molecular projected self-consistent Hamiltonian namely, can reveal the electronic transport of molecular devices. In other words, if orbitals evenly distribute on the space or the extensibility of orbitals throughout the entire molecule, the continuity of orbital may facilitate the electronic transmission and vice versa. By investigate their MPSH energy spectrums, we can see that the HOMO and the LUMO of M1_1_ are nonlocalized (Fig. 4(a) and (d)). For M2_1_, only LUMO is nonlocalized (Fig. 4(e)). However, for M3_1_, both HOMO and LUMO are localized (Fig. 4(c) and (f)). Therefore, the electronic performance of these three models is in the order of M1_1_ > M2_1_ > M3_1_, which agrees with the total electronic conduction relationship described in Fig. 3(b). In general, according to Figs 3(b) and 4, M1_1_ have a bigger electronic conduction than M2_1_ and M3_1_ at the chemical potential where power factor get the maximum value. Nevertheless, spatial distributions of orbitals is just one factor to affect the electronic performance of molecular devices, another is the position of the energy of frontier molecular orbitals in molecular which can be indirectly displayed by the Fig. 3(b). Although the extensibility of the HOMO and LUMO of M2_1_ is better than M3_1_, but as the ZT obtain maximum value the electrical conduction of the devices are affected by the relative position of the molecular orbitals to the chemical potential, which leads to the electrical conduction of M3_1_ being greater than M2_1_.

The chemical potential dependence of ZT for four structures at room temperature is summarized in Fig. 3(d). We note that the ZT can be greatly affected by the change of the contact geometries. The best ZT value of M1_1_ can reach 1.2, while best ZT value of M2_1_ and M3_1_ is 0.1 and 0.4, respectively. This indicates that M1_1_ has the best thermoelectric performance among M1_1_-M3_1_. From Fig. 2(a) and 3(b), although the phonon thermal conductance of M1_1_, M2_1_ and M3_1_ are 3.8 times, 209.9 times and 43.2 times greater, respectively, than electronic thermal conductance, the total thermal conductance of M1_1_, M2_1_ and M3_1_ is 0.15 *nW*/*K*, 0.15 *nW*/*K* and 0.11 *nW*/*K*, respectively. Therefore, the excellent thermoelectric performance of M1_1_ is mainly determined by its electronic conductance, which is induced by the spatial distributions and relative position of their HOMO and LUMO, as mentioned above.

**Figure 5:**
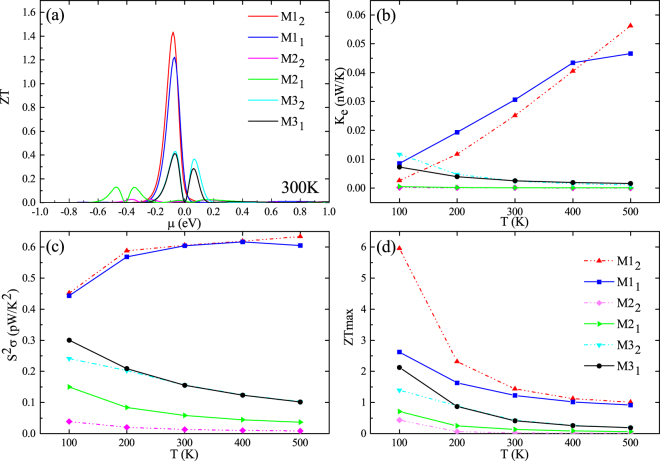
(**a**) Figure of merit ZT of M1_1(2)_-M3_1(2)_ as a function of chemical potential at 300 K. (**b**) Electron thermal conductance, (**c**) power factor and (**d**) ZTmax of M1_1(2)_-M3_1(2)_ as a function of temperature.

**Figure 6:**
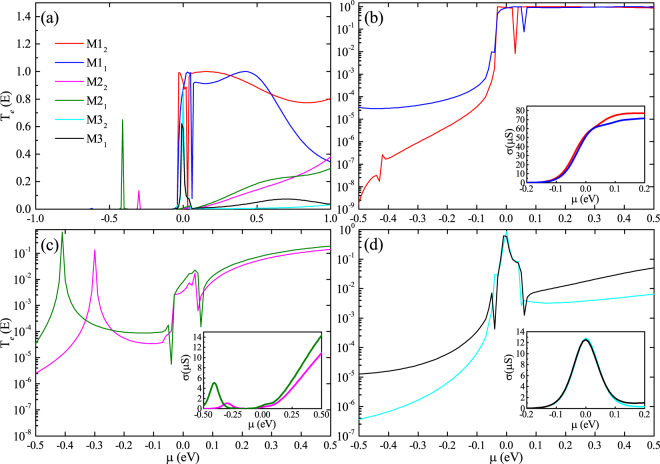
Electronic transmission spectrums of all structures. The insets are the chemical-dependent of electrical conductance.

In order to further improve the figure of merit, we investigated the influence of the coupling between molecule and electrodes to the thermoelectric properties of these molecular devices at different temperatures. Besides, we plot the electronic thermal conduction and the power factor as a function of temperature in Fig. 5(b) and (c), respectively. Contrast to M1_1_, M1_2_ has a lower electron thermal conductance while the power factor is almost unchanged at the temperature of 300 K. According to the formula of ZT, the ZT value will raise with the decrease of the total thermal conductance if other parameters in the formula can almost not changed. By viewing the electronic transmission spectrums of devices at room temperature (Fig. 6), we can know that the transmission spectrum will have a displacement along the energy axis. Considering that the semi-infinite electrode and the central molecule of M1_1 (2)_ have the same configuration, the change of transverse zones, which located on both sides of the central molecule in central region, will affect the coupling between the molecule and the electrodes. Consequently, this displacement is attributing to the changing of the coupling between the molecule and the electrodes. The insets in Fig. 6 also show that the electronic conductance will have the same trend as transmission spectrum. This will lead to a decrease of electronic conductance and electronic thermal conductance of M1. Consequently, the best ZT value of M1_2_ can be enhanced to 1.4 at room temperature (see in Fig. 5(a)). Meanwhile, M2 and M3 are also of the similar phenomenon. However, the difference is that the changes of coupling will make the power factor of M2_2_ and M3_2_ dramatically declined, then the ZT value is also reduced.

We further investigated the maximum ZT value of the six models at different temperature in Fig. 5(d). From the figure, we can obtain that the maximum ZT value of all the structures are decreased as the temperature increases, and the ZT of M1_2_ can reach to 5.9 at 100 K. The reasons for these phenomenon are that although the rise of temperature can make an increase in the value of *σS* ^2^ *T*, it cannot set-off the negative effect, which causing by the remarkable growth of the *κ*_*e*_ + *κ*_*ph*_, leading the maximum ZT reduce as the temperature raise. In addition, the electronic thermal conduction of M1_2_ is about two times smaller than that of M1_1_, making the ZT value of M1_2_ almost improved double times than M1_1_ at 100 K.

## Conclusion

In summary, we investigate the thermoelectric properties of phenalenyl-based single-molecule devices using the non-equilibrium Green’s function method combined with the density functional theory. We find that the thermoelectric properties can be largely regulated by changing the contact geometry of phenalenyl molecule, which is mainly affected by the different of the electronic transport performance. Moreover, the thermoelectric performance of the molecular devices increases rapidly with the decrease of temperature, indicating that these devices are prefer to effectivity use at low temperature and room temperature. In addition, we find that the change of coupling between molecule and electrodes can also enhance ZT value significantly. The results show that M1_1_ and M1_2_ have great potential in thermoelectric application in the future.

## Method

The geometrical optimization of the models and the calculation of force constant matrices are performed by using the Vienna Ab initio simulation package^39, 40^, and the standard of force convergence on each atom is smaller than 0.01 *eV*/$\dot{A}$, which is widely used in previous research^41–43^. Moreover, the phonon-phonon interaction in GNRs has been ignored for the longer phonon mean-free-path^44^. The interaction between nucleus and extra-nuclear electrons has been described with the projector augmented-wave (PAW) pseudopotentials^45^. The specific calculation detail can refer to this work^46^. The electron Hamiltonians and the electron transmission function are obtained by using ATOMISTIX TOOLKIT^47, 48^. The core electrons have been represented with norm-conserving pseudopotentials while the local-density approximation (LDA) has been used as the exchange-correlation potential. The k-point sampling is (1, 1, 100) at the x, y, z direction, and the cutoff energy is set to 150 Ry. A double-zeta polarized (DZP) basis set is used for electron wave function while the convergence criteria of the Hamiltonian and the electron density are 10^−5^. What’s more, the electron-phonon interaction has been neglected due to the weak electron-phonon coupling in GNRs^49^.

In the framework of density-functional perturbation theory (DFPT)^50^, we can get the force constant matrices. Once the force constant matrices *K* are obtained, we use the non-equilibrium Green’s function (NEGF) method and the mass matrices *M* to calculate the retarded surface Green’s function^51^:

1 $${g}_{\alpha }^{r}=[{(\omega +i\eta )}^{2}M-{K}^{\alpha }]$$

where *α* = *L*, *C*, *R* stands for left lead, central region and right lead. Then we can derive the self-energies ${\sum }_{\alpha }^{{\rm{r}}}={V}^{C\alpha }{g}_{\alpha }^{{\rm{r}}}{V}^{\alpha C}$, broaden function ${{\rm{\Gamma }}}_{\alpha }=i({{\rm{\Sigma }}}_{\alpha }^{{\rm{r}}}-{{\rm{\Sigma }}}_{\alpha }^{a})=-2{\rm{I}}{\rm{m}}\,{{\rm{\Sigma }}}_{\alpha }^{{\rm{r}}}$ and retarded Green’s function of the central region ${G}_{C}^{{\rm{r}}}=[{(\omega +i\eta )}^{2}M-{K}^{C}-{\Sigma }_{L}^{{\rm{r}}}-{\Sigma }_{R}^{{\rm{r}}}]$. According to the Caroli formula, the phonon transmission function is calculated as

2 $${T}_{ph}(\omega )=Tr({G}_{{\rm{C}}}^{r}{\Gamma }_{L}{G}_{C}^{a}{\Gamma }_{R})$$

Then the phonon thermal conductance can be calculated by Landau formula^52^:

3 $${\kappa }_{ph}=\frac{1}{2\pi }{\int }_{0}^{\infty }\int d\omega \hslash \omega {T}_{ph}[\omega ][\frac{\partial n(\omega ,T)}{\partial T}]$$

here *n*(*ω*, *T*) is the Bose-Einstein distribution function, $T=\frac{{T}_{L}+{T}_{R}}{2}$ is the average device temperature and *ω* is the frequency of phonons^44^.

For the electron transport, the electron transmission coefficient *T*_*e*_(*E*) can be calculated in a similar way as the thermal transport with the substitutions ${K}_{\alpha }\to {H}_{\alpha }$ and ${\omega }^{2}M\to ES$, where the *H*_*α*_, *E* and *S* are the electronic Hamiltonian, the electron energy and overlap matrix, respectively. Having obtained the *T*_*e*_(*E*) by using density-functional theory (DFT) combined with NEGF, we can then derive the Seebeck coefficient *S*, the electronic conductance *σ*, and the electron thermal conductance *κ*_*e*_ via Lorenz function:

4 $${L}_{n}(\mu ,T)=\frac{2}{h}{\int }_{-\infty }^{\infty }{T}_{e}(E){(E-\mu )}^{n}\frac{-\partial f(E,\mu ,T)}{\partial E}dE,$$

where *f*(*E*, *μ*, *T*) is Fermi-Dirac distribution function at temperature *T* and chemical potential *μ*.

Finally, *σ*, *S* and *κ*_*e*_ can be expressed as

5 $$\sigma (\mu )={e}^{2}{L}_{0}(\mu ,T)$$

6 $$S(\mu ,T)=\frac{1}{eT}\frac{{L}_{1}(\mu ,T)}{{L}_{0}(\mu ,T)}$$

7 $${\kappa }_{e}(\mu ,T)=\frac{1}{T}[{L}_{2}(\mu ,T)-\frac{{{L}_{1}}^{2}(\mu ,T)}{{L}_{0}(\mu ,T)}].$$
